# The Need for Combining Implicit and Explicit Communication in Cooperative Robotic Systems

**DOI:** 10.3389/frobt.2018.00065

**Published:** 2018-06-04

**Authors:** Naomi Gildert, Alan G. Millard, Andrew Pomfret, Jon Timmis

**Affiliations:** ^1^York Robotics Laboratory, Department of Electronic Engineering, University of York, York, United Kingdom; ^2^Department of Electronic Engineering, University of York, York, United Kingdom

**Keywords:** joint action, implicit communication, explicit communication, collaboration, autonomous systems, interaction, robotics

## Abstract

As the number of robots used in warehouses and manufacturing increases, so too does the need for robots to be able to manipulate objects, not only independently, but also in collaboration with humans and other robots. Our ability to effectively coordinate our actions with fellow humans encompasses several behaviours that are collectively referred to as *joint action*, and has inspired advances in human-robot interaction by leveraging our natural ability to interpret implicit cues. However, our capacity to efficiently coordinate on object manipulation tasks remains an advantageous process that is yet to be fully exploited in robotic applications. Humans achieve this form of coordination by combining implicit communication (where information is inferred) and explicit communication (direct communication through an established channel) in varying degrees according to the task at hand. Although these two forms of communication have previously been implemented in robotic systems, no system exists that integrates the two in a task-dependent adaptive manner. In this paper, we review existing work on joint action in human-robot interaction, and analyse the state-of-the-art in robot-robot interaction that could act as a foundation for future cooperative object manipulation approaches. We identify key mechanisms that must be developed in order for robots to collaborate more effectively, with other robots and humans, on object manipulation tasks in shared autonomy spaces.

## Introduction

Humans have evolved as social creatures to coordinate effectively on a variety of tasks, from dancing to carrying a box between two individuals. In the field of psychology, our ability to coordinate with others is referred to as *joint action*, and arises from several underlying mechanisms (detailed in section 2 2). These mechanisms combine to form an advantageous social process, enabling us to work efficiently with others in an adaptive way. Due to our natural capacity to solve various tasks, humans have served as biological inspiration for robotic systems for decades, from the way we walk (Ames, 2014), to how we learn (Degallier et al., 2008). Joint action is yet another valuable human behaviour that can be used as inspiration in robotic cooperative object manipulation tasks.

Effective cooperation and communication between robots is essential for the completion of many tasks, especially in dynamic environments. If joint action were applied to object manipulation in cooperative robotic systems, its advantages—such as increased efficiency in task execution, robustness, and adaptiveness—could also be translated to the robotic implementation. An autonomous cooperation mechanism based on human behaviour would be beneficial for a wide range of applications where robots must effectively cooperate with one another, as well as humans, such as in the growing industry of automated warehouses.

This paper reviews the existing literature surrounding joint action in humans, human-robot interaction, and robot-robot cooperation, and identifies shortcomings that must be addressed before joint action can be fully realised in robotic systems, for tasks like cooperative object manipulation.

## Joint action in humans

Joint action emerges from various underlying mechanisms, including our ability to: direct our attention to the same place as another's, predict future actions of others, and to alter our own actions to compensate for another's by making assumptions about their capabilities (Sebanz et al., 2006). Most of these mechanisms are implicit social processes, where information is inferred from an action rather than directly communicated via speech or codified gestures (usually referred to as explicit communication). For example, Driver et al. (1999) showed that humans often follow the gaze of others, which improves response time in certain tasks, allowing them to pre-empt outcomes. The implicit process of gaze following similarly plays into *joint attention*, in which humans' attention is aligned by attention cues (Feinman et al., 1992).

Humans can also use force as an implicit cue to coordinate their movements when cooperating on a task. Reed et al. (2006) conducted a study where two humans had to cooperate to jointly move a cursor on a screen by sensing the force being exerted on a lever, rather than via verbal communication. The results showed that subjects would non-verbally devise complementary strategies, and completed the task faster when cooperating with each other than when performing the task alone. Sawers et al. (2017) demonstrated that relatively small interaction forces between partners during cooperative physical interactions, such as dancing, can communicate movement goals and act as guiding cues.

In fact, humans frequently use forms of haptic feedback in joint motor tasks to communicate information. Ganesh et al. (2014) showed that physical interaction with an active partner, caused by explicit reactions to behaviours and haptic feedback, allowed an individual to acquire additional information from their partner, which increased task performance. It has also been demonstrated that limb stiffness extracted from haptic feedback can be used to implicitly infer and communicate the intended movement direction of a limb (Mojtahedi et al., 2017b).

Another implicit social process, known as the *Chameleon Effect*, refers to the sociological phenomenon wherein humans imitate each other's movements and gestures while communicating (Chartrand and Bargh, 1999), to the point where it can even interfere with our own task performance (Sebanz et al., 2003). However, Sebanz et al. (2006) showed that when humans perform an action together, such as carrying an object, participants perform complementary actions in order to implicitly align their goals, rather than simply imitating each other. For example, one human will move backwards as the other moves forward. These behaviours are even observed in children as young as 18 months old (Warneken and Tomasello, 2006), indicating that implicit social processes could be intuitive rather than learned.

Some of the processes listed above have suggested how these coordination features can be used in human-robot and robot-robot interactions, particularly in the field of human rehabilitation (Ganesh et al., 2014; Mojtahedi et al., 2017a,b; Sawers et al., 2017). Other processes have already been investigated within the context of human-robot interaction.

## Joint action in human-robot interaction

As robots continue to be developed for operation in shared autonomy spaces, human-robot interaction has become a necessary area of research. In previous work, the underlying mechanisms of joint action have been explored in a variety of applications to improve human-robot interaction. To illustrate this, we review here examples that have investigated gaze following, prediction of future actions, and using force information to coordinate movements in human-robot interaction.

Breazeal et al. (2005) and Li and Zhang (2017) have studied gaze following as an implicit intention cue in the context of human-robot collaborative task performance, and in assistive robots, respectively. Breazeal et al. (2005) found that through non-verbal implicit cues, humans were able to pre-emptively address potential sources of error due to misunderstanding. This reduced the time it took to perform a task, increased efficiency and robustness to error, and increased the transparency and understandability of the robot's internal state.

Wang et al. (2013) created a probabilistic movement model for “intention inference” in human-robot interaction, which mimicked humans' ability to predict future actions of others. The work modelled generative processes of movements that are directed by intention using Bayes' theorem. The system outperformed other existing algorithms that do not model dynamics, and was able to capture the causal relationship between intention and observed movements.

Regarding work exploring force information in human-robot interaction movement coordination, which is necessary for cooperative object manipulation, studies by Magrini et al. (2015) and Rozo et al. (2015) relate more closely to creating a control system that can assure safety in human-robot interaction, rather than exploring the potential benefits of using force as an implicit cue in cooperation between humans and robots.

Crucially, previous research investigating joint action in human-robot interaction, like that of Breazeal et al. (2005) and Li and Zhang (2017), relies on humans' natural ability to communicate implicitly. Humans are able to collaborate more effectively with robots by either their natural implicit cues being interpreted by the robot, or being able to better understand why a robot is behaving as it is. There is a need to investigate robot-robot algorithms that do not rely on a human “expert” in implicit communication in the loop, to allow for the possibility of communication protocols arising that are not inherently human in nature, and to better suit robot-robot interactions where no human expert will be present. While the inspiration for an improved robotic cooperation mechanism may be a human behaviour, it should not be constrained by its human limitations.

## The need for combining implicit and explicit communication

A fundamental facilitator to joint action in humans is the way we communicate with one another using a combination of implicit and explicit communication. For example, when humans cooperate on tasks such as carrying a box together, one might issue a verbal instruction and then apply force to the object to reinforce that instruction. Such tasks also require adaptability, as the most appropriate combination of the two forms of communication depends on the context of the task.

We believe that effective cooperation between robots could arise from focussing on this specific form of human cooperation in object manipulation. In particular, a robot's ability to adaptively change the combination of implicit and explicit communication it is using for any given situation to better suit the task at hand.

This has the potential to be faster and more efficient than current systems that only employ one form of communication. For example, in a situation where a human is carrying a box with another person, a verbal “Stop!” command to communicate an instruction is likely to be interpreted differently depending on whether or not the person shouting also makes an attempt to stop. Using two forms of communication would also be more fault-tolerant, as an adaptive system could enable robots to cope with faults in one of the communication forms by compensating with the other.

The use of robots in manufacturing applications and warehouses, such as Amazon^1^ and Ocado^2^, is rapidly increasing. For these applications to be successful and effective, robots must soon be able to manipulate objects in a range of configurations, either individually or through cooperating with humans and other robots. If we are able to mimic the behaviour exhibited when two humans cooperate on an object manipulation task, it could have wide-ranging benefits in this growing field of application.

Here, we briefly define implicit and explicit communication in their wider contexts, as well as defining them more specifically in the context of this use case.

### Explicit communication

Breazeal et al. (2005) define explicit communication in humans as a deliberate form of communication “where the sender has the goal of sharing specific information with the collocutor.” To account for technical systems and applications, we definite explicit communication to be: *a direct, deliberate form of communication, where there is a clear associated intent for the transmitted information to be received by another agent or system over an established channel*.

In biological examples, explicit communication typically involves verbal communication, such as speech in humans, or vocal calls in animals. Explicit communication can also relate to gestures or body language that have developed an explicit meaning over time, such as the “okay” hand sign. It is important to not mistake non-verbal explicit cues with implicit communication.

In robotic applications, explicit communication relates to the direct transfer of information from one robot to another. This can occur over numerous conventional channels such as Wi-Fi, Bluetooth, or even synthesised speech and voice recognition software.

### Implicit communication

When discussing the interaction between smart environments and humans, Castelfranchi et al. (2012) stated that a form of communication exists outside of direct or explicit communication, which they called “behavioural implicit communication.” This form of communication involves an action (or practical behaviour) representing as a message in itself, rather than a message being conveyed through language or codified gestures (such as a thumbs-up or a head nod).

While explicit communication requires associated intent, implicit communication differs in that pertinent information is independently inferred by another agent or system, rather than transferred purposefully. For example, a robot programmed to observe fellow robots and imitate them can infer information about an observed robot's programmed behaviour in order to copy it, without the observed robot needing to explicitly engage in the interaction (Winfield and Erbas, 2011).

As explained in section 2 2, there are many examples where implicit communication and implicit cues play into joint action and interaction between humans. In the case of cooperative object manipulation, implicit communication could be achieved through methods such as force consensus (Wang and Schwager, 2016), or observation and imitation Winfield and Erbas (2011).

## Force consensus in robot-robot interaction

For cooperative object manipulation applications, which are often tightly coupled, force consensus in particular provides an attractive option for incorporating implicit communication into cooperation mechanisms. Force consensus is a manipulation technique that typically involves the movement or manoeuvring of objects by multiple robots, requiring agents to use force information to reach an agreement on the direction of movement for the object. Existing work that focuses on force consensus in robot-robot applications could provide valuable insights when developing adaptive cooperation mechanisms for shared autonomy spaces. This section explores existing implementations of force consensus in robot-robot applications, where an expert in implicit communication (like a human in human-robot or human-human interactions) is not present.

### Force consensus between two robots

A simple study was conducted by Aiyama et al. (1999) to investigate the use of implicit communication in cooperative transport of an object between two four-legged robots, without any explicit communication transfer between the two agents. This was achieved by applying very simple strategies separately to the front and rear robots, wherein the robots measured the force of the object being applied to their 6-axis force sensors, and changed their behaviour to ensure coordination by comparing the measured values to appropriate thresholds.

The work of Aiyama et al. (1999) was primitive in the sense that neither robot possessed any knowledge of where they were in relation to the goal location, nor did they utilise path planning algorithms that could have aided them in moving an object between two given locations. The robots were also only tested with two objects of similar thickness. As a result of this, although the system provides a mechanism for two robots to cooperate on carrying an object using implicit communication, it is not particularly adaptive.

In more advanced work investigating the same problem, Pereira et al. (2002) developed a methodology in which two robots, both in simulation and real hardware, were able to coordinate their actions when moving a box to a target location using implicit communication.

A follower robot measured the force and torque being applied by the leader onto the object and aimed to apply complementary force and torque to stabilise the object. The control configuration was based on a compliant linkage system that incorporated a *leadership-lending* mechanism, which enabled the two agents to coordinate themselves in an unknown environment where obstacles were present and had to be avoided.

The system's performance in simulation was compared to explicit communication being executed with an increasing number of errors in message transfer, in order to investigate whether implicit communication could be used as a valid means of communication between two robots. Pereira et al. (2002) concluded that although implicit communication could be used in a situation such as this to convey simple information—as results showed that performance of implicit communication was similar to that of using explicit communication in a reliable environment—it might not be possible to convey more complex data through implicit communication channels alone.

The work described above shows that force consensus is a form of implicit communication that can be used in object manipulation tasks, but is not sufficient in isolation. To the best of our knowledge, there exists no work that focuses on using both implicit and explicit communication for object manipulation to exploit the advantages of combining them in adaptive quantities.

### Force consensus in multi-robot systems

In shared autonomy spaces, robots may be required to manipulate large or heavy objects that cannot be handled by two robots, thus necessitating cooperation with numerous robots. Whilst the research field of multi-robot object manipulation is considerably dense, previous work typically does not involve using force information as an implicit form of communication in order to manoeuvre objects. Object manipulation strategies that do not employ force information include caging (Spletzer et al., 2001; Wan et al., 2012), ensemble control techniques (Becker et al., 2013), multi-agent consensus using local communication protocols (Jadbabaie et al., 2003), and leader-follower networks (Ji et al., 2006).

Groß and Dorigo (2004) and Groß et al. (2006) demonstrate that force measurements can be used by robots that either cannot sense, or have no knowledge of, the target destination in an object manipulation task to improve cooperative transport performance. In this sense, the forces exerted by knowledgeable robots implicitly communicates information to ‘blind' robots, which they can then use to adjust their behaviour.

The work described in Wang and Schwager (2016), provides a way for robots to move objects far heavier than themselves using force consensus. A large group of simple robots, whose trajectory is determined by an individual leader robot, share a collective goal of moving an object. The robots reach a consensus and apply the same force on the object in order to overcome the object's static friction and move it. Periodically, the robots measure the force being exerted on the object, and apply a force-updating law that only uses locally known terms to adjust the force being exerted by each individual robot. The force-updating laws are proven to always converge to the force applied by the leader robot, thus providing consensus without communication.

## Conclusions

In this paper we have highlighted the benefits of the human cooperative mechanism referred to as joint action, and reviewed implementations of joint action in the field of human-robot interaction. A new area of focus for the use of joint action in cooperative object manipulation has been identified, and relevant research in robot-robot applications that could contribute to this have also been explored. Table 1 summarises the body of literature that has contributed to this review, from which we have identified the following shortcomings in the field:

- Applications exist that employ implicit and explicit communication in human-robot interaction, but these still rely on a human expert's ability to interpret implicit and non-verbal cues.
- The field has yet to capitalise on humans' ability to cooperate effectively using joint action, specifically the adaptive combination of implicit and explicit communication.

**Table 1:** An overview of existing literature that may underpin future research on joint action in cooperative object manipulation for shared autonomy spaces.

**Category**	**Publications**
Joint action in humans	Feinman et al., 1992; Chartrand and Bargh, 1999; Driver et al., 1999; Tomasello, 2000; Moore and D'Entremont, 2001; Sebanz et al., 2003, 2006; Reed et al., 2006; Warneken and Tomasello, 2006; Ganesh et al., 2014; Sawers et al., 2017; Mojtahedi et al., 2017a,b
Joint action in human robot interaction	Breazeal et al., 2005; Lawitzky et al., 2010; Wang et al., 2013; Magrini et al., 2015; Rozo et al., 2015; Li and Zhang, 2017
Implicit communication in robot-robot applications	Two robots: Aiyama et al., 1999; Pereira et al., 2002
	Multiple robots: Martinoli and Easton, 2003; Groß and Dorigo, 2004; Groß et al., 2006; Ducatelle et al., 2011; Winfield and Erbas, 2011; Wang and Schwager, 2016

*Work exploring the use of explicit communication in robotic systems has been omitted, as explicit communication protocols are so widespread that they are fundamental to most robotics research*.

There is a need to investigate robot-robot joint action that exploits both implicit and explicit communication in cooperation between robots, by mimicking joint action in humans. However, this must be achieved without relying on a human expert in the loop. It is essential that future systems can function autonomously without a human present, but by mimicking human behaviour, it would be possible for humans to also interact with these systems when necessary. An artificial analogue of joint action could not only improve the efficiency of task execution in robot-robot cooperation, but also in shared autonomy situations where a robot must interact with humans as well.

The main focus of future work must be to explore the adaptive balance between implicit and explicit communication, which will vary based on the task at hand. This will provide a new way for robots to cooperate with each other, and humans, more effectively in object manipulation tasks, paving the way for the autonomous factories of tomorrow.

## Author contributions

NG and AM contributed conception and design of the study; NG wrote the first draft of the manuscript; AM, and NG wrote sections of the manuscript. NG, AM, AP, and JT contributed to manuscript revision, read and approved the submitted version.

### Conflict of interest statement

The authors declare that the research was conducted in the absence of any commercial or financial relationships that could be construed as a potential conflict of interest.

## Footnotes

^1^https://www.cnet.com/news/meet-amazons-busiest-employee-the-kiva-robot/ (Accessed: 30/01/2018)

^2^https://www.theengineer.co.uk/grocery-4-0-ocado-reshapes-retail-grocery-with-robotics-and-automation/ (Accessed: 30/01/2018)
